# Interaction of obesity, metabolic syndrome and Framingham risk on steatohepatitis among healthy Taiwanese: population-based nested case-control study

**DOI:** 10.1186/1475-2840-5-12

**Published:** 2006-05-18

**Authors:** Kuo-Liong Chien, Hsiu-Ching Hsu, Chia-Lun Chao, Bai-Chin Lee, Ming-Fong Chen, Yuan-Teh Lee

**Affiliations:** 1 Institute of Preventive Medicine, College of Public Health, National Taiwan University, Taipei, Taiwan; 2 Department of Internal Medicine, National Taiwan University Hospital, Taipei, Taiwan

## Abstract

**Background:**

There have been scant reports on the cumulative effects of atherosclerotic risk factors on steatohepatitis.

**Methods:**

We defined cases of steatohepatitis (n = 124) from one health examination center at National Taiwan University Hospital from January to December 2002. We selected controls, matched by age, gender and drinking status. Metabolic syndrome was defined by the modified ATP-III guidelines. High-dimensional interactions of risk factors for steatohepatitis were evaluated.

**Results:**

Steatohepatitis cases had the highest C-reactive protein, lymphocytes, Framingham scores and predicted coronary risks. The odds ratio (OR) of metabolic syndrome for steatohepatitis was the highest (OR = 9.9), followed by high glucose status (OR = 4.5) and obesity (OR = 3.6). The highest area under the ROC curve was metabolic syndrome (area = 0.80), followed by obesity (0.75) and high glucose level (0.73). Metabolic syndrome was the highest population-attributable risk factor (0.59). Significant interaction was found with a three-factor model, including obesity, metabolic syndrome and Framingham risk status, with lesser average prediction error (22.6%), higher average cross-validation consistency (6.3) and lower average prediction error (24.3%). Compared with persons with no risk factors, OR increased as the number of risk factors increased (OR = 3.0 with one risk factor, 17.5 with two risk factors, 10.8 with three risk factors, respectively).

**Conclusion:**

Metabolic syndrome, inflammation markers and atherosclerotic risk scores are significantly related to steatohepatitis status among the healthy examinee population in Taiwan.

## Background

Nonalcoholic fatty liver disease is prevalent among the general population and can progress to hepatitis and cirrhosis change. The high prevalence rates (up to 20% in the US population) have had a great impact on public health. The clinical syndrome of liver damage can range from steatosis to nonalcoholic steatohepatitis [1]. The cause of steatohepatitis can be due to alcohol abuse, viral infections, autoimmune disorders, genetic dispositions, and among them, insulin resistance syndrome is the factor of greatest association. Insulin resistance syndrome, composed of various metabolic abnormalities such as dyslipidemia, obesity, hypertension and hyperglycemia, is clearly associated with steatohepatitis in previous studies [2,3]. Survey data on the Italian general population clearly shows fatty liver as associated with atherosclerotic risk factors [4]. In the case of 30 subjects with biopsy-proven steatohepatitis along with normal glucose tolerant cases, steatohepatitis was strongly associated with insulin resistance, including glucose and lipid metabolism [5]. Moreover, impaired glucose tolerance is strongly associated with fibrosis and liver cirrhosis [6]. Among metabolic syndrome individuals, insulin resistance is prevalent with steatohepatitis [7]. Among consecutive fatty liver subjects without overt diabetes, metabolic syndrome elicits a 3.2 fold risk for steatohepatitis [8].

Atherosclerotic risk factors are strongly associated with steatohepatitis. Previous studies have shown that steatohepatitis is strongly associated with obesity and very high prevalent rates of steatohepatitis in 32% and 43% of obese men and women, respectively. High triglyceride levels increase steatohepatitis risk by 1.3-fold, low HDL levels increased risk 2.8- and 3-fold, and diabetes increases risk by 1.4- and 3.5-fold in men and women, respectively [1,8]. However, previous studies have not demonstrated the relative importance of various risk factors and possible interaction effects on steatohepatitis.

This study aims to measure metabolic syndrome and its component risks on nonalcoholic fatty liver disease status. We applied the nested case-control design to ascertain study subjects and two control groups from the healthy population, investigating the high-order interaction of risk factors on steatohepatitis.

## Methods

### Study design and subjects

We collected subjects from January 2002 to December 2002 at one health examination center of National Taiwan University Hospital. We collected in total 5,797 adult subjects (41.4% women) from January 2001 to December 2001 from the health-screening program in a tertiary hospital in Taiwan. The protocol for this study was approved by the board of National Taiwan University Hospital, and informed consent was obtained. All participants enrolled voluntarily. This study was of a nested case control design. After excluding those with HBV and HCV viral infection, heavy alcohol drinkers, and those with a medication history of diabetes or hyperlipidemia, we defined the cases of steatohepatitis as follows: NASH (non-alcoholic steatohepatitis) was defined by ultrasound evidence of fatty liver, fatty infiltration, parenchymal liver disease, liver cirrhosis, liver function profile, and alanine tranferase (ALT) more than 1.5 fold (60 IU/mL). We defined two control groups, matched by the age and gender of the subjects. The first control group was defined by fatty liver or filtration, along with an ALT concentration less than 1.5 fold of normal range. The second control group was defined by ultrasound as having no evidence of either fatty liver or infiltration patterns. We eliminated recall bias, yielding an efficient approach with matching confounding factors such as age, sex and drinking status.

Details of medical history such as medication, hospitalization and smoking status were asked in structural questionnaires. Standardized procedures for physical examination, such as anthropometric measures and blood pressure, were performed. Blood pressure was measured by trained medical assistants, with subjects remaining in a resting position. Body mass index (BMI) was calculated as weight (in kilograms)/height (in meters) square.

### Blood sampling and analytic methods

The procedures for blood sampling and analytic methods have been mentioned in a previous paper [9]. In brief, blood samples were collected from each participant in a fasting status for least 12 hours. Serum total cholesterol levels were measured using the CHOD-PAP method (Boehringer Mannheim, Germany). HDL-C was measured following precipitation of apolipoprotein B-containing lipoproteins with phosphotungstic acid and magnesium ions (Boehringer Mannheim, Germany) [10].

Triglyceride concentrations were measured by the GPO-DAOS method (Wako Co., Japan). All of the lipids mentioned above were measured using an Hitachi 7450 automated analyzer (Hitachi, Japan). LDL-C concentrations were calculated using the Friedewald formula [11]. CRP was measured by automated nephelometric immunoassay using a Beckman Array instrument (Beckman Array 360 system, Canada). The peripheral blood cell counts were measured by a blood cell counter (Sysmex Cell Counter NE-8000, TOA Medical Electronics, Co., Ltd., Kobe, Japan). All the measures from both samples were carried out in a single hospital. The coefficient of variation was 5%.

### Definition of binary variables and metabolic syndrome

Smoking status was stratified into never, abstinence and current categories. Metabolic syndrome status was defined by criteria as defined in the Third Adult Treatment Panel of the National Cholesterol Education Program [12], modified for use with BMI data and waist circumference cut points [13]. Therefore, three of following five criteria were grounds for definition: 1) blood pressure of at least 130/85 mmHg or treatment for hypertension; 2) serum triglyceride of at least 150 mg/dL; 3) HDL cholesterol of <40 mg/dL in men and <50 mg/dL in women; 4) fasting glucose of 110 mg/dL or more; and 5) waist circumference > = 90 cm in men, > = 80 in women or BMI of 27 kg/m2 or greater [14]. Framingham risk scores were calculated among the population, and the population was stratified by high-moderate risk (> = 10%) and low risk (<10%) of cardiovascular disease probability for the future 10 years. High CRP was classified as more than 0.30 mg/dL, which was the 75th percentile cut-off value among the study population. Also, high white blood cell counts and lymphocytes were specified by the top quartile in the study population.

### Statistical analysis

We analyzed study subjects and two groups of controls from a cohort of the healthly check-up population. Stratified by these three groups, continuous variables were presented as the mean ± standard deviation, and categorical data were presented as contingency tables. We tested the differences by ANOVA and chi-square tests. Conditional logistic regression was applied to estimate the odds ratios and corresponding 95% confidence intervals [CI] for cases of fatty liver with abnormal liver function. Proportional odds estimation was applied if the assumption was not rejected.

The risk factors for fatty liver with abnormal liver function, after adjusting for age, gender and alcohol drinking status were determined. We estimated the prediction model ability of metabolic syndrome by the area under a receiver operating characteristic (ROC) curve [15] to separate those who were cases from those who were not, i.e. discrimination between case/control status. The area-under-curve statistics showed the probability that the model assigned a high risk to those who were cases than to those who were not. We also compared the areas under two receiver operating curves between genders graphically. Then, we calculated how closely predicted outcomes agreed with actual outcomes by calibration ability. Population attributable risks (PAR) were estimated by total population prevalence and respective odds ratios. PAR described the percentage of the risk reduction if the risk factor was eliminated from the population. This is a useful indicator to evaluate the impact on the overall occurrence of steatohepatitis status [16]. Conditional logistic regression analysis was used to estimate the odds ratio and 95% confidence interval [CI] of various atherosclerotic and inflammatory risk factors to predict steatohepatitis. Because the score test for the proportional odds assumption was not significant in ordinal categorical outcomes (chi-square = 0.003, df = 1, *P *= 0.96), we summarized the endpoints as binary steatohepatitis vs. non-steatohepatitis groups in high ordered interaction effects. We tested with the Hosmer-Lemeshow chi-square statistic [17], dividing the population risk for case status, plotting figures to compare the difference between predicted and observed prevalence. The small chi-square test statistics indicated good calibration, and values exceeding 20 indicated significant lack of calibration.

High-dimensional interactions of various atherosclerotic risk factors were performed by a MDR (multifactor dimensionality reduction) program [18,19]. We included the following binary risk factors into the model: smoking, obesity, high blood pressure, high triglyceride, low HDL, high fasting glucose, metabolic syndrome, high CRP, high lymphocyte, high white cell count, and moderate-high Framingham risk. The above risk factors were highly correlated and interacted with each other. MDR methods can handle high ordered interaction effects. The model with the lowest prediction error and highest cross-validation consistency was selected for each number of factors considered. The reported cross-validation consistency was the number of cross-validation intervals of a particular risk factor combination as chosen by a MDR average across 10 runs. The average classification and prediction errors were the averages across all cross-validation intervals and all runs [19]. Statistical analyses were performed using SAS, version 9.1 (SAS Institute, Inc., Cary, NC) and STATA, version 9 (Stata Corp., College Station, Texas).

## Results

### The distribution of continuous and binary variables, specified by steatohepatitis, fatty liver only and normal groups

We collected a total of 124 subjects with steatohepatitis, matching by individual age, gender status, collecting 124 subjects with fatty liver only (without abnormal liver function) and 124 normal subjects. We found that there were significant differences among these three groups. Subjects with steatohepatitis had the highest body weight, body mass index, fasting glucose, blood pressure, triglyceride, cholesterol and the lowest HDL cholesterol concentrations. Also, steatohepatitis had the highest inflammation markers such as C-reactive protein, lymphocyte values, Framingham scores and predicted coronary risks. The continuous values decreased in the fatty liver group, and the lowest inflammation markers and Framingham risks were in the normal group (Table 1). The distribution of white blood cell counts and granulocytes was not statistically significant among the three groups.

**Figure 1:**
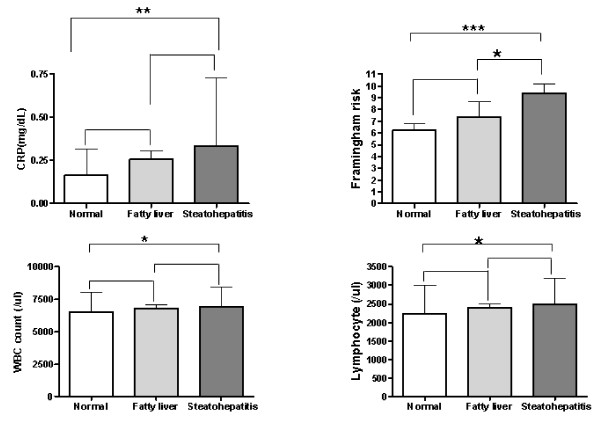
Distribution of inflammation profiles and Framingham coronary risk in the study population, specified by steatohepatitis, fatty liver and normal groups. * ·· P < 0.05 ** ·· P < 0.01 *** ·· P < .001

The frequency distribution of various atherosclerotic risk factors and metabolic syndrome components are described in Table 2. The highest prevalence rates of smoking, hypertension, diabetes, obesity, high blood pressure, hypertriglyceridemia, hyperglycemia, low HDL and metabolic syndrome were found among the subjects with steatohepatitis. Also, the prevalence rates decreased significantly in steatohepatitis, fatty liver and normal groups. For the atherosclerotic and inflammatory risk profiles (Figure 1), we found that Framingham risk, CRP values and lymphocytes counts decreased with steatohepatitis, fatty liver only and the normal pattern.

**Table 1:** Distribution of various atherosclerotic risk factors and inflammation markers, specified by steatohepatitis, fatty liver and normal control groups

			Steatohepatitis			N = 124					Fatty liver only			N = 124					Normal			N = 124			P value*
				
	Mean	Std	median		Q1		Q3		Mean	Std	median		Q1		Q3		Mean	Std	median		Q1		Q3		
Age (years)	49.7 ±	11.0	49	(	42	-	57	)	49.8 ±	10.6	49	(	43	-	57	)	49.5 ±	11.9	49	(	43	-	57	)	0.970
Weight (kg)	77.0 ±	11.4	75	(	70	-	83	)	71.5 ±	10.0	72	(	65	-	78	)	65.0 ±	9.5	65	(	58	-	72	)	<.0001
BMI (kg/m^2^)	27.6 ±	3.1	27	(	25	-	29	)	25.5 ±	2.7	26	(	24	-	27	)	23.4 ±	2.7	23	(	22	-	25	)	<.0001
Fasting glucose (mg/dL)	107.9 ±	29.5	98	(	89	-	117	)	100.1 ±	27.3	92	(	87	-	101	)	92.9 ±	20.8	89	(	84	-	95	)	<.0001
HDL-cholesterol (mg/dL)	42.4 ±	9.2	41	(	36	-	48	)	44.9 ±	10.9	43	(	38	-	49	)	50.9 ±	11.9	50	(	43	-	58	)	<.0001
Triglyceride (mg/dL)	195.9 ±	89.5	176	(	130	-	256	)	166.6 ±	86.0	146	(	115	-	201	)	107.4 ±	51.7	96	(	70	-	136	)	<.0001
Systolic blood pressure (mmHg)**	127.9 ±	13.9	126	(	119	-	137	)	124.5 ±	15.0	123	(	116	-	128	)	120.8 ±	13.6	120	(	110	-	128	)	<.0001
Diastolic blood pressure (mmHg)**	80.7 ±	9.8	80	(	74	-	87	)	77.4 ±	11.7	78	(	71	-	83	)	74.0 ±	11.8	73	(	68	-	82	)	<.0001
Total cholesterol (mg/dL)	202.4 ±	36.0	204	(	183	-	229	)	200.3 ±	33.1	197	(	181	-	217	)	184.4 ±	35.1	182	(	159	-	206	)	<.0001
LDL-cholesterol (mg/dL)	121.3 ±	30.9	119	(	102	-	143	)	122.1 ±	32.2	119	(	105	-	136	)	106.9 ±	29.8	101	(	84	-	125	)	<.0001
White Blood Cell (/μL)	6920 ±	1525	6745	(	5920	-	7960	)	6780 ±	1685	6555	(	5665	-	7685	)	6471 ±	1563	6195	(	5360	-	7900	)	0.071
Lymphocyte (/μL)	2488 ±	693	2442	(	2065	-	2843	)	2384 ±	660	2238	(	1943	-	2753	)	2233 ±	765	2109	(	1674	-	2691	)	0.002
Neutrophils (/μL)	3656 ±	1134	3557	(	2898	-	4445	)	3621 ±	1208	3420	(	2671	-	4186	)	3481 ±	1096	3268	(	2658	-	4183	)	0.371
C-reactive protein (mg/dL)	0.33 ±	0.39	0.24	(	0.14	-	0.37	)	0.26 ±	0.27	0.17	(	0.05	-	0.31	)	0.21 ±	0.32	0.14	(	0.05	-	0.25	)	<.0001
Framingham score	10.9 ±	5.1	11	(	8	-	15	)	9.6 ±	5.1	10	(	7	-	13	)	8.5 ±	5.8	10	(	7	-	12	)	0.006
Framingham risk	9.4 ±	8.6	6	(	3	-	12	)	7.4 ±	7.2	5	(	2	-	10	)	6.3 ±	6.2	4.5	(	1	-	8	)	0.009
Smoking amount (pack*year)	9.7 ±	18.0	0	(	0	-	16	)	5.9 ±	20.2	0	(	0	-	3	)	8.2 ±	15.6	0	(	0	-	12.5	)	0.043
HbA1c (%)	5.9 ±	1.2	5.6	(	5.2	-	6.1	)	5.6 ±	0.9	5.4	(	5.1	-	5.8	)	5.3 ±	0.7	5.1	(	5.0	-	5.4	)	<.0001

*Significance test was by nonparametric Kruskal-Wallis test, Q1: 25th percentile, Q3: 75th percentile, Std: standard deviation

**Table 2:** Distribution of anthropometric, smoking and disease status, specified by steatohepatitis, fatty liver and normal control groups

	**Steatohepatitis**	**N = 124**	**Fatty liver only**	**N = 124**	**Normal**	**N = 124**	**P value**
	n	(%)	n	(%)	n	(%)	

Sex (women)	20	(5.4)	20	(5.4)	20	(5.4)	1.000
Smoking status (yes)	54	(14.5)	37	(10.0)	44	(11.8)	0.078
Hypertension (>=140/90 mmHg or medication)	45	(12.1)	32	(8.6)	22	(5.9)	0.004
Diabetes mellitus (fasting glucose> = 126	23	(6.2)	11	(3.0)	4	(1.1)	0.000
mg/dL or medication)							
Obesity (BMI> = 27)	66	(17.7)	39	(10.5)	10	(2.7)	<.0001
High blood pressure (SBP> = 130 or	61	(16.4)	30	(8.1)	29	(7.8)	<.0001
DBP> = 85 mmHg)							
Triglyceride ≧ 150 mg/dL	77	(20.7)	60	(16.1)	20	(5.4)	<.0001
HDL ≦ 40 (men) or 50 mg/dL (women)	46	(12.4)	33	(8.9)	22	(5.9)	0.003
Fasting glucose ≧ 110 (mg/dL)	40	(10.8)	20	(5.4)	7	(1.9)	<.0001
Metabolic syndrome (Yes)	59	(15.9)	17	(4.6)	6	(1.6)	<.0001

### Relative importance of metabolic syndrome factors for steatohepatitis

To investigate the relative importance of risk factors on steatohepatitis status, we estimated two parameters. The OR of metabolic syndrome status was the highest (OR = 9.9), followed by high glucose status (OR = 4.5) and obesity (OR = 3.6), all statistically significant. Also, the ranking of the area under the ROC curve was similar as OR, the highest with metabolic syndrome (area = 0.80), followed by obesity (area = 0.748) and high glucose level (area = 0.726). Metabolic syndrome was the highest population attributable risk (0.59) among the atherosclerotic risk factors (Table 3).

**Table 3:** Univariate odds ratio*, 95% confidence interval, area under the ROC curve statistics of smoking and metabolic syndrome components for predicting steatohepatitis status

	Odds ratio	95% CI	P value	ROC area	SEM	95% CI	Prevalence rate (%)	PAR
Smoking status (yes)	1.7	(1.1–2.8)	0.030	0.631	0.034	(0.565–0.697)	25.4	0.15
Obesity (BMI> = 27)	3.6	(2.3–5.7)	<.0001	0.748	0.033	(0.685–0.812)	18.0	0.32
High blood pressure (SBP> = 130 or DBP> = 85 mmHg)	3.1	(1.9–5.0)	<.0001	0.706	0.034	(0.640–0.772)	35.4	0.43
Triglyceride ≧ 150 mg/dL	3.3	(2.1–5.2)	<.0001	0.710	0.033	(0.645–0.774)	26.8	0.38
HDL ≦ 40 (men) or 50 mg/dL (women)	2.2	(1.3–3.7)	0.002	0.666	0.036	(0.596–0.736)	37.0	0.31
Fasting glucose ≧ 110 (mg/dL)	4.5	(2.4–8.4)	<.0001	0.726	0.013	(0.699–0.752)	10.9	0.28
Metabolic syndrome (Yes)	9.9	(5.1–19.6)	<.0001	0.800	0.013	(0.774–0.827)	16.3	0.59

*: conditional logistic regression on matched data; CI: confidence interval, ROC: receiver operation characteristics, SEM: standard error of mean

### High order interaction for steatohepatitis status

High-ordered risk factor interaction models predicting steatohepatitis was estimated by an MDR program. For high-order risk factor interactions, MDR showed significant interaction using a three factors model, including obesity, metabolic syndrome and Framingham risk status (Table 4). Compared with other factor combinations, the three risk factor interaction model had the lesser average prediction error (22.6%), the higher average cross-validation consistency (6.3) and the lower average prediction error (24.3%). Also, we found in higher order interaction (> = 6 factors), metabolic syndrome components, instead of metabolic syndrome itself, played a significant role in predicting steatohepatitis (Table 4). We constructed the contingency tables under the final best-fitted three-risk factor model and simplified each risk factor combination into high and low risk groups (Table 5). Comparing subjects with no risk factors, OR increased as the number of risk factors increased from 1 to 3 (OR = 3.0 with one risk factor, 17.5 with two risk factors, 10.8 with three risk factors, respectively).

**Table 4:** High-order Risk Factor Interaction Model in the Study Population

**Risk factor numbers**	**Combination**	**Average classification error (%)**	**Average prediction error (%)**	**Average cross-validation consistency**
1-factor	metabolic syndrome	23.8	23.8	10.0
2-factors	Smoking, metabolic syndrome	23.8	23.8	10.0
3-factors	Obesity, metabolic syndrome, Framingham risk	22.6	24.3	6.3
4-factors	Smoking, obesity, metabolic syndrome, Framingham risk	21.9	26.5	3.4
5-factors	Smoking, obesity, high glucose, metabolic syndrome, Framingham risk	20.6	27.3	3.5
6-factors	Smoking, obesity, high BP, High TG, low HDL, high CRP	18.9	29.2	3.6
7-factors	Smoking, obesity, high BP, high TG, low HDL, high CRP, high lymphocyte	16.4	29.9	4.1
8-factors	Smoking, obesity, high BP, high TG, low HDL, high glucose, high CRP, Framingham risk	14.2	27.3	6.6

**Table 5:** Distribution of binary risk factors in the study population with three risk factor combinations, odds ratios and 95% confidence intervals for steatohepatitis status

**Number of Risk Factors**	**Steatohepatitis (-)**	**Steatohepatitis (+)**	**Odds ratio**	**Lower 95% C.I.**	**Upper 95% C.I.**	**P value**
0	160	32	1.00 (baseline)			
1	66	35	3.0	1.6	5.6	0.000
2	11	36	17.5	7.1	43.2	<.0001
3	11	21	10.8	4.3	27.4	<.0001

Risk group defined by obesity, metabolic syndrome, and high Framingham coronary risk status.

## Discussion

This is the first report on the high order interaction effects of steatohepatitis, constructed with a nested case-control design for the healthy population. We clearly demonstrated obesity, metabolic syndrome, and high coronary risk had interaction effects on steatohepatitis status.

The close relationship between insulin resistance and nonalcoholic fatty liver disease was demonstrated in a spectrum of abnormalities, from fatty liver only to nonalcoholic steatohepatitis. The nonalcoholic steatohepatitis is a critical link in the chain of metabolic fatty liver disorders that spans steatosis to cryptogenic cirrhosis. It is the hepatic manifestation of the insulin resistance (or metabolic) syndrome, and provides a clue to understanding fibrotic progression of other chronic liver diseases, particularly hepatitis C. Nonalcoholic steatohepatitis is often the first clinical indication of insulin resistance, with its complications of high blood pressure, coronary heart disease and type 2 diabetes[20]. Our study clearly demonstrated the atherosclerotic risk burdens in nonalcoholic steatohepatitis.

Abdominal ultrasound is a useful tool to detect fatty liver disease, although with limited sensitivity and specificity. In this nested case-control design, non-invasive ultrasound as the diagnostic tool provided valid and reproducible instruments, which was more effective than the "gold standard" of liver biopsy. Also, the consistency of information collection and blinding in exposure and disease ascertainment made the results valid.

There have been abundant reports in the literature on the association of steatohepatitis and metabolic syndrome [21,22]. For example, a cross-sectional study based on adults demonstrated clearly that metabolic syndrome is independently associated with fatty liver [21]. In Taiwan, obesity and atherosclerotic risk factors play important roles in metabolic syndrome and cardiovascular burdens [23,24]. Our study results imply that the burdens of obesity to the liver are also significant. In the severely obese patients, the best therapeutic modality is bariatric surgery, which is safe and has been successful in producing a considerable weight loss. This treatment can improve the control of diabetes mellitus, the metabolic syndrome, and presumably its sequelae. The bariatric surgery can reduce the fat, inflammation, and even the fibrosis in well-documented nonalcoholic steatohepatitis[25].

Inflammation risk worsens steatohepatitis status and has incremental interactions with metabolic syndrome and risk profiles. Possible mechanisms of inflammatory cytokine increases might play roles in the steatohepatitis process. Our study demonstrates that CRP and lymphocytes were associated with steatohepatitis and fatty liver status. Anti-inflammatory effects of pharmacotherapy, such as insulin sensitizers, might play a role in the reduction of fatty liver and steatohepatitis severity.

The limitations of this study are as follows: First, we cannot specify the accuracy of ultrasound or biopsy evidence for fatty liver. Histopathological features of steatohepatitis have been the gold standard for diagnosis of steatohepatitis. Also, the misclassification of disease and control status might underestimate the true association effects. Due to large study populations and reliable abdominal sonography, the diagnosis of cases and careful selection of controls could validate the results. Second, the cases of steatohepatitis were prevalent cases, not incident cases. The associated factors might have been beneficial for survival status. We included the close population in fixed duration to improve the comparability of case and control status, and the strategy was able to decrease selection bias. The prospective cohort or randomized control trial studies would provide more evidence for risk factor reduction for steatohepatitis control. Finally, the study population was from the specified health examination center, and possible self-selection characteristics among healthy subjects could make external generalization of the results to the general population limited.

## Conclusion

Metabolic syndrome and atherosclerotic risk are strongly associated with steatohepatitis and fatty liver disease status in healthy Chinese. Obesity, metabolic syndrome and high Framingham risk have significant interaction effects on steatohepatitis among people in the healthy population.

## Competing interests

The author(s) declare that they have no competing interests.

## Authors' contributions

KLC carried out data collection, statistical analyses and participated in the study design and processing data. YTL participated in the design of the study and supervised the ideas developed in hypothesis generation. MFC & HCH performed the laboratory measurements for lipid levels and were in charge of quality control. CLC and BCL carried out data collection. All authors read and approved the final manuscript.
